# The effects of mitral stenosis on right ventricular mechanics assessed by three-dimensional echocardiography

**DOI:** 10.1038/s41598-024-68126-y

**Published:** 2024-07-24

**Authors:** Zsuzsanna Ladányi, Abdalla Eltayeb, Alexandra Fábián, Adrienn Ujvári, Máté Tolvaj, Márton Tokodi, Kashif Anwar Choudhary, Attila Kovács, Béla Merkely, Olga Vriz, Bálint Károly Lakatos

**Affiliations:** 1 Heart and Vascular Center, Semmelweis University, Varosmajor Utca 68, Budapest, 1122 Hungary; 2 King Faisal Specialist Hospital and Research Center Hospital, Riyadh, Saudi Arabia; 3 Department of Surgical Research and Techniques, Semmelweis University, Budapest, Hungary; 4 Ospedale Sant’Antonio, San Daniele del Friuli, Italy

**Keywords:** Mitral stenosis, Three-dimensional echocardiography, Right ventricle, Speckle-tracking echocardiography, Contraction pattern, Atrial fibrillation, Cardiology, Cardiovascular biology, Cardiovascular diseases, Valvular disease

## Abstract

Mitral stenosis (MS) is a complex valvular pathology with significant clinical burden even today. Its effect on the right heart is often overlooked, despite it playing a considerable part in the symptomatic status. We enrolled 39 mitral valve stenosis patients and 39 age- and gender-matched healthy controls. They underwent conventional, speckle-tracking and 3D echocardiographic examinations. The 3D data was analyzed using the ReVISION software to calculate RV functional parameters. In the MS group, 3D RV ejection fraction (EF) (49 ± 7% vs. 61 ± 4%; *p* < 0.001), global circumferential (GCS) (− 21.08 ± 5.64% vs. − 25.07 ± 4.72%; *p* = 0.001) and longitudinal strain (GLS) (− 16.60% ± 4.07% vs. − 23.32 ± 2.82%; *p* < 0.001) were reduced. When comparing RV contraction patterns between controls, MS patients in sinus rhythm and those with atrial fibrillation, radial (REF) (32.06 ± 5.33% vs. 23.62 ± 7.95% vs. 20.89 ± 6.92%; *p* < 0.001) and longitudinal ejection fraction (LEF) (24.85 ± 4.06%; 17.82 ± 6.16% vs. 15.91 ± 4.09%; *p* < 0.001) were decreased in both MS groups compared to controls; however, they were comparable between the two MS subgroups. Anteroposterior ejection fraction (AEF) (29.16 ± 4.60% vs. 30.87 ± 7.71% vs. 21.48 ± 6.15%; *p* < 0.001) showed no difference between controls and MS patients in sinus rhythm, while it was lower in the MS group with atrial fibrillation. Therefore, utilizing 3D echocardiography, we found distinct morphological and functional alterations of the RV in MS patients.

## Introduction

While the prevalence of mitral stenosis (MS) has greatly decreased in industrialized countries, it remains a significant healthcare problem in developing countries and affects young patients, lingering as a heavy clinical burden even nowadays^1,2^. One of the most important etiological factors of MS is rheumatic valve disease, which is still prevalent in developing countries, while degenerative MS constantly increases within the aging population of the developed nations^3–7^. The timing of therapeutic interventions is a delicate issue, and many patients receive treatment in a suboptimally late stage of the disease.

Left ventricular (LV) systolic function is usually preserved in cases of isolated MS. As the valve progressively narrows, the cardiac output becomes subnormal at rest and fails to increase during exercise. More importantly, in parallel with the worsening mitral transvalvular gradient, left atrial and pulmonary pressures increase, posing a significantly higher load to the right ventricle (RV). Beyond the cardioembolic events in association with the left atrial dilation and blood stasis, pulmonary congestion and RV dysfunction are the main factors of morbidity and mortality^8^. Notably, tricuspid regurgitation due to the primary rheumatic involvement of the valve and/or secondary functional origin also contributes to the RV adverse remodeling^2,9^.

While the effects of MS on the left heart and the lungs have been extensively studied, its influence on the right heart is often overlooked despite playing a significant part in the symptomatic status and outcome of the patients. Current guidelines recommend intervention in the case of significant pulmonary hypertension; however, RV remodeling and dysfunction in MS patients are scarcely studied, especially using state-of-the-art cardiovascular imaging methods^2^. Therefore, we aimed to examine the RV morphology and functional status of MS patients using three-dimensional (3D) echocardiography, focusing on the differences in 3D RV ejection fraction (EF), strain values and the absolute and relative contribution of the different RV motion components.

## Methods

### Study population

We prospectively enrolled 39 MS patients between 2019 and 2022 and at the King Faisal Specialist Hospital and Research Center Hospital (Riyadh, Saudi Arabia). The study was approved by the Research Ethics Committee and Research Affairs Office of King Faisal Specialist Hospital and Research Center Hospital (IRB number 2201042) and all of the subjects have provided a written informed consent to the study procedures. Every study participant was more than 18 years of age at the time of enrollment. Inclusion criteria was the presence of at least moderate rheumatic MS. Exclusion criteria were a history of previous cardiac surgeries, degenerative MS, congenital heart disease or other valvular disease (except for mild mitral regurgitation and secondary tricuspid regurgitation of any severity). An age-, sex and body surface area (BSA)-matched population recruited from a community screening program at Semmelweis University Heart and Vascular Center (Budapest, Hungary) served as a control group (CTR). CTR subjects also provided written informed consent to the examinations and the study was approved by the Semmelweis University Regional and Institutional Committee of Science and Research Ethics (IRB number 169/2018). Control patients did not have a history and/or symptoms of any cardiovascular or pulmonary disease and did not have any cardiovascular risk factors such as arterial hypertension, diabetes, smoking, and dyslipidemia. Exclusion criteria were the presence of any abnormality on electrocardiography, moderate or severe valvular heart disease or wall motion abnormality and/or LV EF < 50% found during echocardiography, poor echocardiographic windows, and factors that might affect cardiac morphology and function, such as pregnancy and regular high-intensity sport activity (> 3 h/week). Detailed medical history and symptomatic status were obtained, and all patients underwent conventional, speckle-tracking and 3D echocardiographic examinations.

### Conventional echocardiography and left ventricular speckle tracking analysis

Conventional echocardiographic evaluations were conducted utilizing a commercially available ultrasound system (E95, GE Healthcare, Horten, Norway) equipped with dedicated software for 3D RV evaluation, and a matrix-array transducer (3.5 MHz). A standard acquisition protocol encompassing parasternal, apical, and subxiphoid views was used in accordance with the current guidelines^10^. LV end-diastolic (EDVi) and the end-systolic (ESVi) volume indices were quantified utilizing the biplane Simpson method, indexed to the body surface area (BSA). Continous-wave Doppler imaging was used to measure MV mean gradient. For mitral valve area (MVA) planimetry, 3D-based approach was used by creating a cutting plane on the parasternal long axis view at the level of the maximum opening, then MVA was measured in the corresponding short axis view. Tricuspid annular plane systolic excursion (TAPSE) was determined using M-mode imaging, calculated as the maximum longitudinal displacement of the tricuspid annulus. The maximum velocity of the tricuspid regurgitant jet (TR V_max_) was calculated using continuous-wave Doppler imaging. Peak pulmonary artery systolic pressure (PASP) was calculated based on the velocity of the tricuspid regurgitant jet and the estimated right atrial pressure, which was deduced from inferior vena cava diameter and collapsibility. Atrial volumes were estimated using the Simpson method, and by indexing both to BSA, left atrial volume index (LAVi) and right atrial volume index (RAVi) were determined. Tricuspid regurgitation was quantified according to current guidelines^2^. RV basal diameter was measured in apical 4-chamber view. Fractional area change (FAC) was assessed by contouring the end-diastolic (RV EDA) and end-systolic RV endocardial areas (RV ESA).

LV speckle-tracking analysis was performed during post-processing using the dedicated module of a commercially available software solution (EchoPAC v204, GE Healthcare, Horten, Norway). The software automatically identified apical four-, three- and two-chamber views, and semi-automatically applied the corresponding region of interest. Recordings with a lower frame rate than 50 FPS were excluded (none). Two-dimensional LV global longitudinal strain (GLS) values were assessed by speckle-tracking echocardiography. Acceptance or rejection of a particular segment was guided by the software’s recommendation. Global longitudinal strain was not noted in the case of three or more rejected segments (none).

### Three-dimensional echocardiography

In addition to the standard echocardiographic protocol, electrocardiogram (ECG)-gated full-volume 3D datasets were obtained. Multi beat acquisition was strongly preferred, 3D data was reconstructed from either four or six cardiac cycles and tailored to optimize visualization of the RV. In the case of significant beat-to-beat heart cycle variation, single beat acquisition was used. Image quality assessment was performed at the patient’s bedside to mitigate potential “stitching” and “dropout” artifacts. All measurements were performed during post-processing in an offline fashion by an experienced operator (LBK), blinded to the study groups. Subsequent analyses were conducted utilizing dedicated software (4D RV-Function 2; TomTec Imaging, Unterschleissheim, Germany). This software possesses the capability to automatically identify the endocardial surface of both the LV and RV; manual adjustments were performed if necessary to ensure accurate tracking of ventricular motion throughout the cardiac cycle.

**Figure 1:**
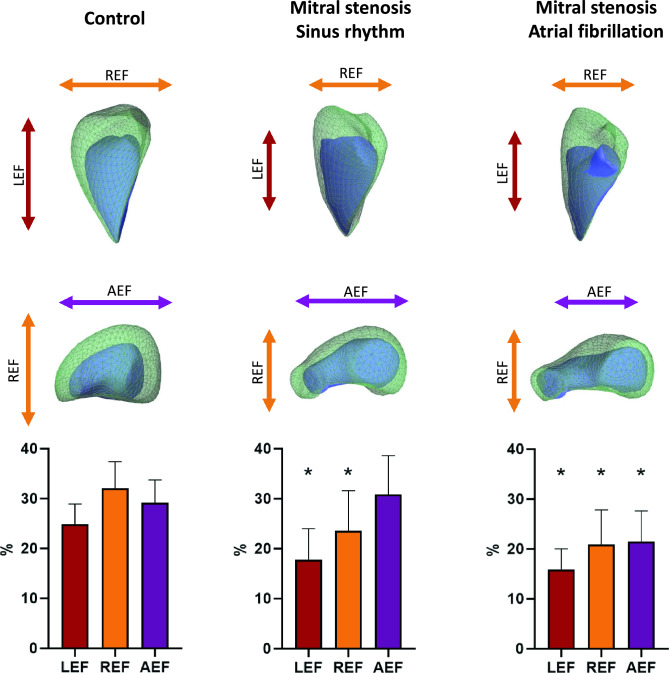
Graphical representation of a healthy control, a mitral stenosis patient in sinus rhythm and a mitral stenosis patient with atrial fibrillation in terms of three-dimensional right ventricular volumes and mechanics. The mitral stenosis patients in sinus rhythm (MS-SR) and the mitral stenosis patients with atrial fibrillation (MS-AF) had smaller end-diastolic volumes than the controls (CTR), while the MS-AF patients had larger end-systolic volumes than both the CTR and MS-SR patients (green mesh—right ventricular end-diastolic volume; blue surface—right ventricular end-systolic volume). Concerning right ventricular motion decomposition, both MS groups had lower LEF and REF values than the CTR group, whereas AEF remained comparable between the CTR and MS-SR patients, while it was lower in the MS-AF group. *LEF*, longitudinal ejection fraction; *REF*, radial ejection fraction; *AEF*, anteroposterior ejection fraction; *, significant difference compared to healthy contols.

3D RV deformation analysis was performed using commercially available software (ReVISION, Argus Cognitive, Lebanon, New Hampshire), with the methodology already described in detail^11,12^, and validated on a large cohort^13^. First, the 3D mesh model exported from the TomTec 4D RV-Function software package was re-oriented by a standard, automated method to identify the longitudinal (from the tricuspid annulus to the apex), radial (perpendicular to the interventricular septum), and anteroposterior (parallel to the interventricular septum) axes. Next, motion decomposition was performed along these directions in a vertex-based manner to quantify component values generated by each motion component [i. e. longitudinal EF (LEF), radial EF (REF), and anteroposterior EF (AEF)], as described in previous methodology papers. Our results regarding these motion components are visualized in Fig. 1. To comprehensively evaluate global RV function, EF was also calculated. The relative contribution of each component to the total RV pump function was expressed as the ratio between LEF, REF, and AEF and total RV EF (LEF/RV EF, REF/RV EF, and AEF/RV EF, respectively). Various other cardiac parameters were also assessed: 3D EDVi, ESVi, and stroke volume index (SVi), all of which were normalized to BSA. We evaluated RV global circumferential strain (RV GCS) and RV global longitudinal strain (RV GLS). Moreover, regional strain values were also determined, including septal circumferential strain (SCS), septal longitudinal strain (SLS), free wall circumferential strain (FWCS) and free wall longitudinal strain (FWLS).

### Statistical analysis

We performed statistical analysis using STATISTICA version 13.4 (TIBCO Software Inc, Palo Alto, CA, USA) and GraphPad Prism 8.0.1 version (GraphPad Software Inc, San Diego, CA, USA). We performed a power analysis to define the statistical power and the optimal sample size. Using a relevant earlier study of the field^14^ we determined the effect-sizes (Cohen’s d) of representative parameters that describe RV morphology and function (RV EDD; RV FAC; TAPSE). After calculating Cohen’s d, all of these parameters are considered to have an adequate effect size (Cohen’s d values respectively: 0.883; 0.667; 1.265). Using these calculated effect sizes, we performed power analysis on the retrospectively identified subjects from our database. In all cases the statistical power exceeded 80%, reassuring that the outlined sample size that we proposed for our current study was appropriate. We verified the normal distribution of our variables using the Shapiro–Wilk test. Data are presented as mean ± standard deviation, median (interquartile ranges), or number of patients (percentage), as appropriate. For continuous variables, the data of the MS and the control patients were compared using the unpaired Student’s t-test or Mann–Whitney U test; and for categorical variables, the chi-square or Fisher’s exact test according to normality. The control group and the MS patients in sinus rhythm (SR) and with atrial fibrillation (AF) were compared using the ANOVA test. For post-hoc analysis, Fisher’s least significant difference (LSD) test was implemented. Correlations were analyzed using Pearson or Spearman correlation, as appropriate. Chi-square test was used to compare the severity of tricuspid regurgitation in MS patient in SR and with AF. *P*-values < 0.05 were considered statistically significant.

We assessed intra- and interobserver variability of the most relevant parameters. The operator of the first measurements (B.K.L.) and a second expert reader (Z.L.) repeated the measurements on a randomly chosen subset of 5–5 MS and control patients. Both operators reconstructed the 3D RV models again, then the fully automated ReVISION method was applied to the 3D models, and reproducibility of ESV values with either only longitudinal, only radial or only anteroposterior motion component enabled was calculated. Decomposed ESV values, paired with the EDV value, are markers of the interreader variability of the motion components and the contouring as well, therefore, in the publications using the ReVISION method^15–17^, decomposed ESV values are used for inter- and intraobserver variability. As the ReVISION method is a fully automated technique, it adds no further variability on top of the commercially available software for 3D model reconstruction. Lin’s concordance correlation coefficient and coefficient of variation were calculated.

### Ethical approval

The study was performed in accordance with the Declaration of Helsinki and GCP. Written informed consent and assent were obtained from all participants.

## Results

### Patient characteristics

The baseline characteristics of the patients are presented in Table 1.

**Table 1:** Baseline characteristics of the patients.

	Mitral stenosis	Control	*P*-value
	N = 39	N = 39	
Age (years)	54 ± 9	55 ± 9	0.714
Female (n)	34 (87%)	32 (82%)	0.530
Height (cm)	157 ± 8	163 ± 7	**0.001**
Weight (kg)	73 ± 18	67 ± 11	0.089
BSA (m^2^)	1.72 ± 0.21	1.74 ± 0.17	0.666
SBP (mmHg)	121 ± 14	138 ± 21	**< 0.001**
DBP (mmHg)	73 ± 10	82 ± 13	**0.002**
HR (1/min)	71 ± 14	75 ± 13	0.139
NYHA stage 2 + (n)	3	–	
Hypertension (n)	9	–	
Diabetes mellitus (n)	11	–	
Atrial fibrillation (n)	18 (46%)	–	

Data are presented as mean ± SD or number of patients (%).

BSA, body surface area; SBP, systolic blood pressure; DBP, diastolic blood pressure; HR, heart rate; NYHA, New York Heart Association.

Values with a significant difference are presented in bold.

The MS population was 54 ± 9 years of age, and 34 (87%) of them were female. Every patient had at least moderate MS, along with numerous other comorbidities. The selected control group was age- and gender-matched [55 ± 9 years of age, and 32 (82%) female]. The MS cohort had lower height, while there was no difference in their weight and BSA compared to the controls. Systolic and diastolic blood pressures were higher in the control group.

### Conventional echocardiographic parameters

The results of the conventional echocardiographic examinations can be found in Table 2.

**Table 2:** Conventional echocardiographic parameters of the mitral stenosis and the control groups.

	Mitral stenosis	Control	*P*-value
	N = 39	N = 39	
LV EDVi (ml/m^2^)	49.38 ± 13.47	53.64 ± 11.24	0.133
LV ESVi (ml/m^2^)	20.80 ± 7.27	21.33 ± 5.55	0.721
LV EF (%)	58.3 ± 8.1	61.0 ± 4.6	0.068
LV GLS (%)	− 18.1 ± 3.1	− 18.4 ± 2.9	0.670
MV area (cm^2^)	1.41 ± 0.29		
MV mean gradient (mmHg)	7.63 ± 4.06		
LAVi (ml/m^2^)	63.74 ± 31.03	29.76 ± 7.31	**< 0.001**
RAVi (ml/m^2^)	25.91 ± 14.13	27.96 ± 8.16	0.435
LVIDD (mm)	46 ± 5	43 ± 4	**0.039**
RV basal diameter (mm)	36 ± 6	30 ± 4	**< 0.001**
RV/LV ratio	0.78 ± 0.20	0.69 ± 0.09	**0.011**
TAPSE (mm)	19 ± 5	24 ± 4	**< 0.001**
TR V_max_ (cm/s)	2.66 ± 0.49	2.18 ± 0.24	**< 0.001**
PASP (mmHg)	36 ± 12	23 ± 4	**< 0.001**
RV EDA (cm^2^)	17 ± 4	25 ± 6	**< 0.001**
RV ESA (cm^2^)	10 ± 2	14 ± 5	**< 0.001**
FAC (%)	39.2 ± 8.7	43.3 ± 4.7	**0.013**
Tricuspid S’ (cm/s)	10 ± 3	14 ± 2	**< 0.001**

Data are presented as mean ± SD.

LV, left ventricular; EDVi, end-diastolic volume index; ESVi, end-systolic volume index; EF, ejection fraction; GLS, global longitudinal strain; MV, mitral valve; LAVi, left atrial volume index; RAVi, right atrial volume index; LVIDD, left ventricular internal diameter in end-diastole; TAPSE, tricuspid annular plane systolic excursion; TR, tricuspid valve regurgitation; PASP, pulmonary artery systolic pressure; RV EDA, right ventricular end-diastolic area; RV ESA, right ventricular end-systolic area; FAC, fractional area change.

Values with a significant difference are presented in bold.

There was no difference in left ventricular morphology or function between the two groups. LAVi was significantly larger in MS compared to the controls.

Regarding the right heart, the MS group had higher PASP, lower TAPSE and larger RV basal diameter than the controls. RV EDA, RV ESA and FAC were all smaller in MS patients. Moreover, TR V_max_ was higher in the MS group.

### 3D right ventricular systolic function

The data of the global and segmental 3D RV deformation measures are presented in Table 3.

**Table 3:** Comparison of 3D right ventricular data measured by the ReVISION method in the mitral stenosis and the control groups.

	Mitral stenosis	Control	P-value
	N = 39	N = 39	
RV EDVi (ml/m^2^)	46.92 ± 11.63	53.47 ± 10.60	**0.011**
RV ESVi (ml/m^2^)	23.88 ± 7.15	20.88 ± 5.09	**0.036**
RV SVi (ml/m^2^)	23.04 ± 6.37	32.59 ± 6.38	**< 0.001**
RV EF (%)	49.24 ± 7.33	61.13 ± 3.87	**< 0.001**
RV GCS (%)	− 21.08 ± 5.64	− 25.07 ± 4.72	**0.001**
RV GLS (%)	− 16.60 ± 4.07	− 23.32 ± 2.82	**< 0.001**
REF (%)	22.36 ± 7.52	32.06 ± 5.33	**< 0.001**
REF/RV EF	0.45 ± 0.13	0.52 ± 0.07	**0.002**
AEF (%)	26.54 ± 8.41	29.16 ± 4.60	0.091
AEF/RV EF	0.53 ± 0.12	0.48 ± 0.06	**0.013**
LEF (%)	16.94 ± 5.33	24.85 ± 4.06	**< 0.001**
LEF/RV EF	0.34 ± 0.10	0.41 ± 0.06	**0.001**
SCS (%)	− 16.99 ± 6.67	− 18.33 ± 5.84	0.350
SLS (%)	− 13.64 ± 5.55	− 20.47 ± 4.14	**< 0.001**
FWCS (%)	− 21.08 ± 5.62	− 25.21 ± 4.85	**0.001**
FWLS (%)	− 20.04 ± 5.16	− 26.89 ± 4.41	**< 0.001**

Data are presented as mean ± SD.

RV, right ventricle; EDVi, end-diastolic volume index; ESVi, end-systolic volume index; SVi, stroke volume index; EF, ejection fraction; GCS, global circumferential strain, GLS, longitudinal strain; REF, radial ejection fraction; AEF, anteroposterior ejection fraction; LEF, longitudinal ejection fraction; SCS, septal circumferential strain; SLS, septal longitudinal strain; FWCS, free wall circumferential strain; FWLS, free wall longitudinal strain.

Values with a significant difference are presented in bold.

Regarding RV morphology and systolic function, RV EDVi was lower, while RV ESVi was higher than in the controls, resulting in a lower RV stroke volume index (SVi) in MS patients. RV EF, GCS and GLS were all lower in the MS group compared to the controls.

Concerning the decomposed RV motion components and the relative contribution of longitudinal, radial, and anteroposterior wall motions to global RV function, REF was lower in the MS population and so was REF/RV EF. AEF showed no significant difference between the two groups, however, AEF/RV EF was higher in MS patients. LEF and LEF / RV EF were both lower in the MS group.

Regarding the septal and free wall function, there was no change in SCS, however, SLS was also lower. FWCS and FWLS were also significantly lower in the MS groups.

We found a number of correlations between the 3D RV echocardiographic measures and the conventional parameters of RV function, as well as LV GLS, however, there was no association between the 3D parameters and the severity of MS (Supplementary Table 1).

### The effect of atrial fibrillation

We assessed the differences between the controls (CTR group) MS patients with SR (MS-SR group) and AF (MS-AF group) (Table 4).

**Table 4:** The comparison of the controls and the mitral stenosis patients with sinus rhythm vs. with atrial fibrillation.

	Control	Mitral stenosis –Sinus rhythm	Mitral stenosis—Atrial fibrillation	*P*-value
	N = 39	n = 21	N = 18	
LV EDVi (ml/m^2^)	53.64 ± 11.24	49.68 ± 15.71	49.02 ± 10.73	0.321
LV ESVi (ml/m^2^)	21.33 ± 5.55	20.65 ± 7.82	20.98 ± 6.79	0.927
LV EF (%)	61.0 ± 4.6	59 ± 8	58 ± 8	0.164
LV GLS (%)	− 18.4 ± 2.9	− 18.7 ± 3.3	− 16.8 ± 2.0	0.227
MV area (cm^2^)	–	1.42 ± 0.31	1.39 ± 0.28	0.739
MV mean gradient (mmHg)	–	8.44 ± 4.06	6.68 ± 3.97	0.181
LAVi (ml/m^2^)	29.76 ± 7.31 *#	54.44 ± 17.33 †#	74.68 ± 39.69 †*	**< 0.001**
RAVi (ml/m^2^)	27.96 ± 8.16 *#	16.49 ± 8.50 †#	36.90 ± 11.11 †*	**< 0.001**
LVIDD (mm)	44 ± 4	46 ± 5	46 ± 5	0.131
RV basal diameter (mm)	30 ± 4 *#	35 ± 6 †	37 ± 6 †	** < 0.001**
RV/LV ratio	0.70 ± 0.10	0.78 ± 0.11	0.79 ± 0.27	0.105
TAPSE (mm)	24 ± 4 *#	21 ± 4 †#	17 ± 5 †*	**< 0.001**
PASP (mmHg)	23 ± 4 *#	34 ± 9 †	38 ± 14 †	**0.001**
RV EDA (cm^2^)	25 ± 6 *#	16 ± 4 †	17 ± 3 †	**< 0.001**
RV ESA (cm^2^)	14 ± 5 *#	9 ± 2 †	11 ± 2 †	**< 0.001**
FAC (%)	43.3 ± 4.7 #	41.8 ± 8.4 #	36.3 ± 8.4 †*	**0.002**
Tricuspid S’ (cm/s)	14 ± 3 *#	12 ± 3 †#	9 ± 2 †*	**< 0.001**
RV EDVi (ml/m^2^)	53.47 ± 10.60 *#	45.65 ± 9.62 †	48.41 ± 13.75 †	**0.031**
RV ESVi (ml/m^2^)	20.88 ± 5.09 #	21.96 ± 6.18 #	26.12 ± 7.70 †*	**0.012**
RV SVi (ml/m^2^)	32.59 ± 6.38 *#	23.69 ± 5.62 †	20.29 ± 7.24 †	**< 0.001**
RV EF (%)	61.13 ± 3.87 *#	52.10 ± 7.25 †#	45.90 ± 6.03 †*	**< 0.001**
RV GCS (%)	− 25.07 ± 4.72 #	− 23.43 ± 5.66 #	− 18.33 ± 4.31 †*	**< 0.001**
RV GLS (%)	− 23.32 ± 2.82 *#	− 17.97 ± 3.96 †#	− 15.01 ± 3.69 †*	**< 0.001**
REF	32.06 ± 5.33 *#	23.62 ± 7.95 †	20.89 ± 6.92 †	**< 0.001**
REF / RV EF	0.52 ± 0.07 *#	0.45 ± 0.12 †	0.45 ± 0.14 †	**0.007**
AEF	29.16 ± 4.60 #	30.87 ± 7.71 #	21.48 ± 6.15 †*	**< 0.001**
AEF / RV EF	0.48 ± 0.06 *	0.59 ± 0.10 †#	0.47 ± 0.12 *	**< 0.001**
LEF	24.85 ± 4.06 *#	17.82 ± 6.16 †	15.91 ± 4.09 †	**< 0.001**
LEF / RV EF	0.41 ± 0.06 *#	0.34 ± 0.12 †	0.35 ± 0.08 †	**0.006**
SCS (%)	− 18.33 ± 5.84 #	− 19.22 ± 5.77 #	− 14.39 ± 6.85 †*	**0.034**
SLS (%)	− 20.47 ± 4.14 *#	− 14.04 ± 5.94 †	− 13.18 ± 5.19 †	**< 0.001**
FWCS (%)	− 25.21 ± 4.85 #	− 23.40 ± 5.67 #	− 18.38 ± 4.30 †*	**< 0.001**
FWLS (%)	− 26.89 ± 4.41 *#	− 21.98 ± 4.62 †#	− 17.77 ± 4.92 †*	**< 0.001**

Data are presented as mean ± SD.

LV = left ventricular, EDVi = end-diastolic volume index, ESVi = end-systolic volume index, EF = ejection fraction, GLS = global longitudinal strain, MV = mitral valve, LAVi = left atrial volume index, RAVi = right atrial volume index, LVIDD = left ventricular internal diameter in end-diastole, TAPSE = tricuspid annular plane systolic excursion, PASP = pulmonary artery systolic pressure, TR = tricuspid valve regurgitation, RV EDA = right ventricular end-diastolic area, RV ESA = right ventricular end-systolic area, FAC = fractional area change, RV = right ventricle, EF = ejection fraction; GCS = global circumferential strain, GLS = longitudinal strain; EDVi = end-diastolic volume index; ESVi = end-systolic volume index; SVi = stroke volume index; REF = radial ejection fraction; AEF = anteroposterior ejection fraction; LEF = longitudinal ejection fraction; SEF = septal ejection fraction; SCS = septal circumferential strain; SLS = septal longitudinal strain; FWEF = free wall ejection fraction; FWCS = free wall circumferential strain; FWLS = free wall longitudinal strain.

*p < 0.05 vs. Mitral stenosis—Sinus rhythm; #*p* < 0.05 vs. Mitral stenosis—Atrial fibrillation; †*p* < 0.05 vs. Control.

Values with a significant difference are presented in bold.

As expected, the MS-AF group had significantly larger LAVi, and also RAVi. The MS-SR and MS-AF groups had higher PASP than the CTR group. The MS-SR group had lower TAPSE, RV EF and GLS than the CTR patients, while the MS-AF group had even lower values. FAC and RV GCS were comparable between the CTR and MS-SR groups, while the MS-AF patients had lower values.

**Figure 2:**
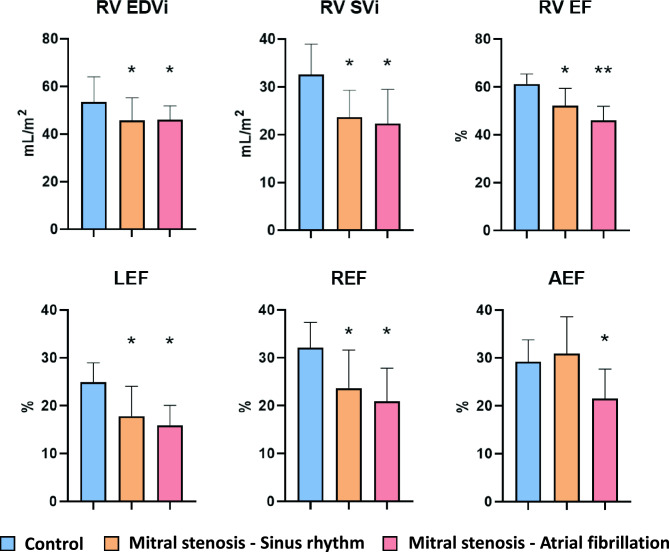
The comparison of right ventricular morphological and functional parameters in healthy controls, mitral stenosis patients in sinus rhythm and mitral stenosis patients with atrial fibrillation. Both the mitral stenosis patients in sinus rhythm (MS-SR) and the mitral stenosis patients with atrial fibrillation (MS-AF) had lower RV EDVi and RV SVi compared to healthy controls (CTR). The MS-SR group had lower RV-EF compared to the CTR group, while the MS-AF patients presented with even lower values. LEF and REF were lower in both the MS-SR and the MS-AF groups compared to the CTR group, while AEF was lower only in the MS-AEF group. *RV EDVi*, right ventricular end-diastolic volume index; *RV SVi*, right ventricular stroke volume index; *RV EF*, right ventricular ejection fraction; *LEF*, longitudinal ejection fraction; *REF*, radial ejection fraction; *AEF*, anteroposterior ejection fraction; *, significant difference compared to healthy contols, **, significant difference compared to healthy contols and mitral stenosis patients in sinus rhythm.

Interestingly, when comparing motion components, REF and LEF were both significantly lower in both MS groups than in CTR patients, while these parameters were comparable between the MS-SR and MS-AF groups. On the other hand, AEF was comparable between the CTR and the MS-SR groups, while the MS-AF patients had significantly lower values (Figs. 1 and 2).

When comparing septal and free wall function, both SCS and FWCS were comparable between the controls and the MS groups, while the MS-AF patients presented with lower values. However, SLS and FWLS were lower in both MS groups than in the CTR group, and the MS-AF patients had even lower FWLS values than the MS-SR group.

Moreover, AF patients had generally higher severity of tricuspid regurgitation (Supplementary Table 2).

### Inter- and intraobserver variability

The intra- and interreader variability analysis of our key parameters showed good agreement in the evaluation of these measures (Table 5).

**Table 5:** Inter- and intraobserver variability.

	Intraobserver variability		Interobserver variability	
	ICC	CV	ICC	CV
RV EDV	0.966	3.840	0.992	2.502
RV EF	0.965	1.767	0.941	2.941
Radial ESV	0.935	8.921	0.934	8.032
Anteroposterior ESV	0.860	10.180	0.833	12.962
Longitudinal ESV	0.965	4.789	0.926	10.287

ICC, intraclass correlation coefficient; CV, coefficient of variation; RV, right ventricular, EDV, end-diastolic volume; EF, ejection fraction; ESV, end-systolic volume.

## Discussion

Conventional echocardiography provides a wide array of LV functional parameters^18^. However, regarding the RV, clinicians mainly examine the longitudinal component of systolic function using 2D echocardiography, which is severely limited in its capability of estimating global RV function^19^. While 3D echocardiography gained popularity in recent years, previously there was no dedicated method to explore both global and regional 3D RV deformation and the relative contribution of the different RV motion components (e.g. longitudinal, radial and anteroposterior) to global RV EF. Accordingly, this is the first study assessing 3D global and regional RV mechanics in MS patients, which may hold additional value in this complex population. Based on our findings: i) MS patients had lower 3D global RV EF, GLS and GCS values compared to age- and sex-matched controls; ii) regarding the relative contribution to global RV EF, the longitudinal and radial components of RV wall motion were reduced in MS, whereas the anteroposterior component had a higher contribution to the global RV EF in MS patients than in controls; iii) MS patients had decreased RV septal and free-wall 3D strain values, except for septal circumferential strain, which was comparable between the two groups; iv) AF mainly deteriorated those motion components (anteroposterior ejection fraction and septal circumferential strain) that were maintained when comparing pooled MS patients compared with healthy controls, resulting in the worsening of the global RV function.

MS is a valvular heart disease that clinically presents as chronically compensated LV backward failure, therefore, the prevalence of LV dysfunction in MS is controversial; studies report about 30% of incidence; however, many of the manuscripts are quite dated^20–24^. Accordingly, it is no surprise that there was no LV morphological or functional difference between the two cohorts in our study population.

While MS is a valvular disease of the left heart, it predominantly poses hemodynamic overload to the pulmonary circulation and consequently, to the right side of the heart. Moreover, AF, a common complication of MS, may facilitate these adverse effects. Therefore, it is of great importance to investigate the RV function in MS patients. Nevertheless, routinely used measures of RV morphology and function disregard the complex shape and functional characteristics of the ventricle. 3D RV assessment provides added clinical value in a wide variety of diseases^25^, however, these metrics have not been investigated in MS.

Considering that backward effects dominate the clinical signs of MS, the increased left atrial pressure results in left atrial dilatation^26^, and thus, increased LAVi, which was a prognostic marker of adverse cardiac events in patients with progressive MS in a study, where LAVi was mainly influenced by the presence of AF and the severity of MS^27^.

As the backward failure progresses, pulmonary congestion may occur, marked by an increased PASP^28^, as seen in our cohort. PASP at peak exercise is a predictor of clinical outcomes in MS patients and adds incremental prognostic value beyond what can be provided by standard resting measurements, including valve area^29^. According to current guidelines, markedly elevated PASP (> 50 mmHg) is also an indication for intervention in asymptomatic rheumatic MS patients^2^. Interestingly, PASP did not show a relationship with MS severity markers or RV functional measures, suggesting that the RV can effectively compensate for the corresponding hemodynamic overload, and in MS, other factors also significantly influence RV mechanics.

Nevertheless, MS in the late stages commonly affects the RV, leading to dilation and the possible impairment of RV function. In previous studies, severe rheumatic MS patients presented with preserved RV FAC and relatively normal TAPSE; nevertheless, these values were decreased compared to healthy controls^30^, with significant improvement seen at 6 months after percutaneous mitral commissurotomy^31^. However, TAPSE has several limits as it is measured in a single direction, is angle-dependent, and entirely ignores the septal contribution to the RV function^19^. Speckle-tracking analysis can provide a more detailed view on RV functional status, unveiling subclinical stages of myocardial dysfunction. Previously in MS patients 2D RV strain measures were found to be altered before clinical signs of systemic venous congestion presented^32–34^. Decreased 2D RV GLS and FWLS in patients with MS are associated with higher rate of rehospitalization and morbidity^35^. Although, 2D strain parameters are less load-dependent than conventional measures of RV function, they still explore the deformation of the myocardium in limited directions, mostly focusing on longitudinal motion and only quantifying the function of the RV inflow tract in a single tomographic plane. Apart from decreased longitudinal function, impaired radial motion is also common in MS, as in a previous study, although within the normal range, FAC was also lower in MS patients than in healthy controls^30^. FAC also suffers from the inherent 2D nature of its calculation, referring to a single plane of the large RV wall surface. Therefore, neither of these parameters reflect RV function precisely due to the complex shape of the chamber. 3D RV measures may mitigate this limitation, as they may show RV dysfunction before the apparent impairment of traditional parameters in various other clinical scenarios, highlighting their additional value^13,16,17,25,36–39^. In our study, 3D RV EF was lower in the MS cohort than in the healthy controls, albeit still within normal range, while 3D RV GLS and GCS were both decreased. Regarding RV motion decomposition, radial and longitudinal functions were decreased in the MS group, while anteroposterior function remained unchanged. These findings can also explain the mildly lower TAPSE and FAC usually seen in MS patients, while the EF generally remains within the normal spectrum. Due to these changes, while radial and longitudinal motion contributed less to the global RV function, the anteroposterior motion had a higher impact on global RV contraction in the MS population, playing a significant part in trying to maintain the RV function. Similarly to our observations, previous experimental studies with normal and mechanically unloaded (sudden impairment in preload) LV contractions showed markedly increased anteroposterior shortening of the RV during unloaded LV beats^40^. Interestingly, when comparing septal and free wall motion, septal circumferential strain remained unchanged, while all other strain values decreased. The reason behind this probably lies in the interdependence of the ventricles, the maintained LV function compensating through the shared circumferential myofilaments of the interventricular septum^41,42^.

Apart from the pressure overload caused by MS, AF is also known to have a negative effect on RV function, as in a previous MS study, patients with AF had lower baseline 2D strain values than SR patients^31^. TAPSE was lower in both the MS-SR and MS-AF groups compared to healthy controls, albeit not dramatically deteriorated in either MS group. Of note, FAC remained comparable with MS-SR and control patients. However, the RV dysfunction caused by AF was more prominent when comparing 3D functional measures. Global and free wall systolic functions were decreased, while in the septum, the previously unaltered circumferential strain decreased significantly in AF. Similarly, it was the anteroposterior function (which was comparable between the MS and the healthy control populations) that decreased in AF patients. Notably, AF patients had generally higher severity of tricuspid regurgitation, which may contribute to this finding. Moreover, AF may significantly deteriorate RV motion components by substantially impairing RV diastolic filling, that were responsible for compensating the global RV function in the MS patients, resulting in the 3D RV EF being decreased in a considerable proportion of MS patients having AF.

### Limitations

Our study has a number of limitations that should be acknowledged for adequate interpretation. Firstly, this study enrolled a limited number of patients—further expansion of the population, especially in a multicentre setting, would strengthen our findings, and sub-group analysis is limited in such a small population. Secondly, systolic and diastolic blood pressures were higher in the control group; patients with high ambulatory blood pressure were further examined to exclude hypertension, they underwent further visits and 24 h blood pressure measurement, and thus, the possible diagnosis of hypertension was excluded in all cases, and the high initial blood pressure values were considered to be the result of white coat hypertension; however, even white coat hypertension can be a cardiovascular risk factor. Thirdly, this is a cross-sectional study, and the association of advanced 3D RV parameters with outcome was not evaluated. Further downstream exams and additional clinical data recording were not included in the study protocol and IRB, which focused on the echocardiographic data; which is a limiting factor. Furthermore, 3D echocardiographic acquisition requires expert training, moreover, it is not yet a part of the clinical routine. Additionally, in patients with AF the single beat method was used for 3D acquisition, which is a limitation due to its lower temporal and spatial resolution; however, the single beat approach shows significantly lower variability in AF than the multibeat approach^43^. Also, further studies with larger cohorts and longer follow-up periods are needed to underpin the prognostic value of the abnormal RV parameters in MS patients.

### Conclusions

We found distinct morphological and functional alterations in the RV using 3D echocardiography, while the LV function remained unchanged in MS patients. We examined the changes in RV motion components previously undescribed in this population. Nonetheless, the underlying pathophysiological basis and clinical significance of our observations regarding 3D RV parameters remain unclear and necessitate further research. Notwithstanding this, our data substantiate the use of comprehensive echocardiographic protocol incorporating advanced methodologies in the management of MS patients.

### Supplementary Information

Supplementary Tables.

## Data Availability

The datasets presented in this article are not readily available due to patient data privacy regulations. Requests to access the datasets should be directed to the corresponding author and have to be authorized by the local Data Management Committee.
