# Supplementary material for: The effects of mitral stenosis on right ventricular mechanics assessed by three-dimensional echocardiography
Source: Sci Rep. 2024 Jul 24;14:17112. doi: 10.1038/s41598-024-68126-y (PMC11269591; doi:10.1038/s41598-024-68126-y)
Supplement: Supplementary file 1 — Supplementary Tables. [file 41598_2024_68126_MOESM1_ESM.pdf]

# The effects of mitral stenosis on right ventricular mechanics assessed by three-dimensional echocardiography

Zsuzsanna Ladányi [1*], Abdalla Eltayeb [2], Alexandra Fábián [1], Adrienn Ujvári [1], Máté Tolvaj [1], Márton Tokodi [1,3], Kashif Anwar Choudhary [2], Attila Kovács [1,3], Béla Merkely [1], Olga Vriz [2,4], Bálint Károly Lakatos [1]

[1] Heart and Vascular Center, Semmelweis University, Budapest, Hungary

[2] King Faisal Specialist Hospital and Research Center Hospital, Riyadh, Saudi Arabia

[3] Department of Surgical Research and Techniques, Semmelweis University, Budapest, Hungary

[4] Ospedale Sant'Antonio, San Daniele del Friuli, Italy

*** Correspondence:**

**Zsuzsanna Ladányi MD**

email: lazsuzs9@gmail.com

phone: +3614586810

address: Varosmajor utca 68, Budapest, Hungary 1122

Supplementary materials

**Supplementary Table 1: Correlations of 3D right ventricular echocardiographic measures with conventional parameters**

| | **Age (years)** | **LVEDVi (ml)** | **LVESVi (ml)** | **LVEF (%)** | **LVGLS (%)** | **LAVi ($ml/m^2$)** | **MV area ($cm^2$)** | **Mean mitral valve gradient (mmHg)** | **PASP (mmHg)** | **TAPSE (mm)** | **FAC (%)** | **RAVi ($ml/m^2$)** |
|---|---|---|---|---|---|---|---|---|---|---|---|---|
| **RV EF (%)** | r=0.155<br>p=0.345 | r=0.152<br>p=0.357 | r=0.046<br>p=0.779 | r=0.132<br>p=0.425 | **r=-0.631<br>p<0.001** | r=-0.094<br>p=0.582 | r=0.294<br>p=0.069 | r=-0.279<br>p=0.086 | r=-0.180<br>p=0.287 | **r=0.442<br>p=0.005** | **r=0.549<br>p<0.001** | **r=-0.332<br>p=0.039** |
| **RV GCS (%)** | r=-0.080<br>p=0.630 | r=-0.092<br>p=0.578 | r=-0.096<br>p=0.561 | r=0.025<br>p=0.880 | **r=0.633<br>p<0.001** | r=0.123<br>p=0.470 | r=-0.144<br>p=0.381 | r=0.134<br>p=0.415 | r=0.119<br>p=0.482 | **r=-0.366<br>p=0.024** | **r=-0.432<br>p=0.006** | r=0.298<br>p=0.065 |
| **RV GLS (%)** | r=-0.068<br>p=0.681 | r=-0.281<br>p=0.083 | r=-0.197<br>p=0.229 | r=-0.026<br>p=0.878 | r=0.165<br>p=0.374 | r=-0.083<br>p=0.626 | r=-0.241<br>p=0.139 | r=0.156<br>p=0.344 | r=0.159<br>p=0.347 | **r=-0.325<br>p=0.047** | **r=-0.463<br>p=0.003** | **r=0.389<br>p=0.014** |
| **RV EDVi (ml)** | r=-0.027<br>p=0.869 | r=0.276<br>p=0.089 | r=0.235<br>p=0.150 | r=-0.025<br>p=0.882 | r=-0.106<br>p=0.570 | r=0.137<br>p=0.419 | r=0.173<br>p=0.292 | r=0.084<br>p=0.612 | r=0.325<br>p=0.050 | r=0.109<br>p=0.515 | r=0.068<br>p=0.683 | **r=0.450<br>p=0.004** |
| **RV ESVi (ml)** | r=-0.099<br>p=0.551 | r=0.147<br>p=0.373 | r=0.156<br>p=0.342 | r=-0.062<br>p=0.708 | r=0.175<br>p=0.348 | r=0.162<br>p=0.338 | r=0.001<br>p=0.997 | r=0.209<br>p=0.203 | **r=0.370<br>p=0.024** | r=-0.101<br>p=0.547 | r=-0.203<br>p=0.216 | **r=0.548<br>p<0.001** |
| **RV SVi (ml)** | r=0.061<br>p=0.714 | **r=0.339<br>p=0.035** | r=0.254<br>p=0.119 | r=0.025<br>p=0.882 | **r=-0.430<br>p=0.016** | r=0.062<br>p=0.716 | r=0.315<br>p=0.051 | r=-0.081<br>p=0.624 | r=0.181<br>p=0.285 | r=0.313<br>p=0.056 | **r=0.351<br>p=0.029** | r=0.207<br>p=0.206 |
| **REF (%)** | r=0.291<br>p=0.072 | r=0.155<br>p=0.346 | r=0.085<br>p=0.608 | r=0.060<br>p=0.716 | **r=-0.529<br>p=0.002** | r=-0.079<br>p=0.643 | r=0.082<br>p=0.618 | r=-0.257<br>p=0.115 | r=-0.002<br>p=0.990 | **r=0.329<br>p=0.043** | **r=0.348<br>p=0.030** | r=-0.058<br>p=0.726 |
| **REF/ RVEF** | **r=0.347<br>p=0.031** | r=0.130<br>p=0.431 | r=0.098<br>p=0.552 | r=-0.003<br>p=0.987 | **r=-0.334<br>p=0.067** | r=-0.024<br>p=0.890 | r=-0.085<br>p=0.607 | r=-0.178<br>p=0.279 | r=0.108<br>p=0.526 | r=0.164<br>p=0.324 | r=0.155<br>p=0.347 | r=0.148<br>p=0.368 |
| **AEF (%)** | r=-0.129<br>p=0.433 | r=0.094<br>p=0.569 | r=-0.007<br>p=0.967 | r=0.116<br>p=0.481 | **r=-0.635<br>p<0.001** | r=-0.156<br>p=0.355 | r=0.242<br>p=0.138 | r=0.005<br>p=0.974 | r=-0.107<br>p=0.530 | **r=0.351<br>p=0.031** | **r=0.458<br>p=0.003** | **r=-0.445<br>p=0.005** |
| **AEF/ RVEF** | r=-0.294<br>p=0.069 | r=0.005<br>p=0.977 | r=-0.060<br>p=0.719 | r=0.078<br>p=0.637 | **r=-0.438<br>p=0.014** | r=-0.144<br>p=0.395 | r=0.152<br>p=0.357 | r=0.188<br>p=0.253 | r=-0.047<br>p=0.785 | r=0.190<br>p=0.254 | r=0.269<br>p=0.098 | **r=-0.421<br>p=0.008** |
| **LEF (%)** | r=0.149<br>p=0.365 | r=0.170<br>p=0.300 | r=0.135<br>p=0.413 | r=0.015<br>p=0.928 | r=0.130<br>p=0.487 | r=0.071<br>p=0.678 | r=0.226<br>p=0.166 | r=-0.195<br>p=0.235 | r=-0.262<br>p=0.117 | r=0.287<br>p=0.081 | r=0.240<br>p=0.141 | r=-0.255<br>p=0.118 |
| **LEF/ RVEF** | r=0.063<br>p=0.704 | r=0.087<br>p=0.597 | r=0.117<br>p=0.479 | r=-0.064<br>p=0.700 | **r=0.443<br>p=0.013** | r=0.099<br>p=0.559 | r=0.101<br>p=0.543 | r=-0.097<br>p=0.557 | r=-0.238<br>p=0.156 | r=0.087<br>p=0.603 | r=-0.029<br>p=0.860 | r=-0.109<br>p=0.508 |
| **SCS (%)** | r=0.202<br>p=0.217 | r=0.074<br>p=0.653 | r=-0.002<br>p=0.988 | r=0.064<br>p=0.699 | **r=0.371<br>p=0.040** | r=0.070<br>p=0.681 | r=-0.056<br>p=0.737 | r=-0.014<br>p=0.932 | r=0.183<br>p=0.280 | r=-0.210<br>p=0.206 | r=-0.106<br>p=0.523 | r=0.238<br>p=0.145 |
| **SLS (%)** | r=0.184<br>p=0.263 | r=-0.115<br>p=0.486 | r=-0.057<br>p=0.733 | r=-0.072<br>p=0.663 | r=-0.238<br>p=0.198 | r=-0.144<br>p=0.397 | r=-0.169<br>p=0.304 | r=0.116<br>p=0.481 | r=0.187<br>p=0.268 | r=-0.020<br>p=0.905 | r=-0.275<br>p=0.090 | r=0.150<br>p=0.362 |
| **FWCS (%)** | r=-0.107<br>p=0.518 | r=-0.078<br>p=0.639 | r=-0.108<br>p=0.511 | r=0.058<br>p=0.724 | **r=0.587<br>p=0.001** | r=0.114<br>p=0.501 | r=-0.106<br>p=0.521 | r=0.159<br>p=0.332 | r=0.142<br>p=0.403 | **r=-0.348<br>p=0.032** | **r=-0.425<br>p=0.007** | r=0.286<br>p=0.077 |
| **FWLS (%)** | r=0.004<br>p=0.980 | r=-0.308<br>p=0.056 | r=-0.172<br>p=0.294 | r=-0.093<br>p=0.572 | r=0.217<br>p=0.242 | r=-0.065<br>p=0.701 | r=-0.179<br>p=0.277 | r=0.028<br>p=0.864 | r=0.105<br>p=0.536 | r=-0.302<br>p=0.066 | **r=-0.474<br>p=0.002** | **r=0.372<br>p=0.020** |

Values with a significant correlation are presented in bold.

Abbreviations: LV = left ventricular, EDVi = end-diastolic volume index, ESVi = end-systolic volume index, EF = ejection fraction, GLS = global longitudinal strain, MV = mitral valve, LAVi = left atrial volume index, RAVi = right atrial volume index, TAPSE = tricuspid annular plane systolic excursion, PASP = pulmonary artery systolic pressure, TR = tricuspid valve regurgitation, RVAD = right ventricular area in diastole, RVAS = right ventricular area in systole, FAC = fractional area change, RV = right ventricle, EF = ejection fraction; GCS = global circumferential strain, GLS = longitudinal strain; EDVi = end-diastolic volume index; ESVi = end-systolic volume index; SVi = stroke volume index; REF = radial ejection fraction; AEF = anteroposterior ejection fraction; LEF = longitudinal ejection fraction; SEF = septal ejection fraction; SCS = septal circumferential strain; SLS = septal longitudinal strain; FWEF = free wall ejection fraction; FWCS = free wall circumferential strain; FWLS = free wall longitudinal strain

**Supplementary Table 2: The severity of tricuspid regurgitation in mitral stenosis patients with atrial fibrillation vs. in sinus rhythm**

| | No TR | Mild TR | Moderate TR | Severe TR |
|---|---|---|---|---|
| **Sinus rhythm** | 2 (5%) | 15 (38%) | 4 (10%) | 0 (0%) |
| **Arial fibrillation** | 0 (0%) | 7 (18%) | 7 (18%) | 4 (10%) |

**p for trend = 0.02**

Data is presented as number of patients (%).

Abbreviations: TR = tricuspid regurgitation
